# Population Pharmacokinetics and Initial Dose Optimization of Sirolimus Improving Drug Blood Level for Seizure Control in Pediatric Patients With Tuberous Sclerosis Complex

**DOI:** 10.3389/fphar.2021.647232

**Published:** 2021-04-20

**Authors:** Xiao Chen, Dongdong Wang, Lin Zhu, Jinmiao Lu, Yidie Huang, Guangfei Wang, Yiqing Zhu, Qiaofeng Ye, Yi Wang, Hong Xu, Zhiping Li

**Affiliations:** ^1^ Department of Pharmacy, Children’s Hospital of Fudan University, Shanghai, China; ^2^ Department of Nephrology, Children’s Hospital of Fudan University, Shanghai, China; ^3^ Department of Neurology, Children’s Hospital of Fudan University, Shanghai, China

**Keywords:** population pharmacokinetics, initial dose optimization, sirolimus, seizure control, tuberous sclerosis complex

## Abstract

The purposes of this study were to explore the population pharmacokinetics and initial dose optimization of sirolimus improving drug blood level for seizure control in pediatric patients with tuberous sclerosis complex (TSC). Eighty pediatric patients diagnosed with TSC-related epilepsy were included for analysis. Sirolimus concentrations, physiological and biochemical indexes, and drug combination were collected to build a nonlinear mixed effect (NONMEM) model. Initial dose optimization was simulated by the Monte Carlo method. The weight and concomitant medication of oxcarbazepine affected sirolimus clearance. Without oxcarbazepine, for once-daily sirolimus regimen, the doses of 0.07, 0.06, 0.05, 0.04, and 0.03 mg/kg/day were recommended for weights of 5–7.5, 7.5–11.5, 11.5–19, 19–40, and 40–70 kg, respectively; for twice-daily sirolimus regimen, the doses of 0.05, 0.04, and 0.03 were recommended for weights of 5–8, 8–20, and 20–70 kg, respectively. With oxcarbazepine, for once-daily sirolimus regimen, the doses of 0.09, 0.08, 0.07, 0.06, 0.05, and 0.04 mg/kg/day were recommended for weights of 5–7.5, 7.5–10, 10–13.5, 13.5–20, 20–35, and 35–70 kg, respectively; for twice-daily sirolimus regimen, the doses of 0.06, 0.05, 0.04, and 0.03 were recommended for weights of 5–7, 7–14.5, 14.5–38, and 38–70 kg, respectively. The present study was the first to establish a population pharmacokinetic model of sirolimus improving drug blood level for seizure control in pediatric patients with TSC and recommend the initial dosage regimen.

## Introduction

Tuberous sclerosis complex (TSC), is an autosomal dominant neurodermal syndrome, most of which are caused by abnormal organ development in the ectodermal tissue, involving multiple organs such as the brain, the skin, the peripheral nerves, and the kidney, and leading to the mammalian target of rapamycin (mTOR) pathway hyperactivation (Curatolo et al., 2008; van der Poest Clement et al., 2020). Its clinical features include epileptic seizures, facial angiofibroma, and decreased intelligence, among them, epilepsy is the most common neurologic complication and influences about 90% of patients, in which 2/3 are drug resistant (Chu-Shore et al., 2010; Kwan et al., 2010). If the seizure is not well controlled, it will seriously affect children’s intellectual development, reduce their quality of life, and increase the burden of the whole society (He et al., 2020). For the time being, the available treatments include a ketogenic diet, antiepileptic drugs, vagus nerve stimulation, and epilepsy surgery (He et al., 2020). However, the therapeutic outcomes of these treatments for individuals with TSC patients, in general, are not satisfactory (Curatolo et al., 2015; de Vries et al., 2015).

Sirolimus is an mTOR inhibitor and has been widely proved that it has good effects on treating many of the symptoms of TSC (Bissler et al., 2008; Davies et al., 2011; Nathan et al., 2015; Wataya-Kaneda et al., 2017). Moreover, He *et al* reported that sirolimus has a significant effect on seizures associated with TSC, and it could be used as the first-line medication for pediatric patients with TSC-associated epilepsy (He et al., 2020). However, sirolimus has a narrow therapeutic window with considerable inter- and intra-individual pharmacokinetic variabilities (Wang et al., 2020), making it difficult to design a sirolimus initial dosage regimen, especially for sirolimus improving drug blood level for seizure control in pediatric patients with TSC. Therefore, the purposes of this study were to explore the population pharmacokinetics and initial dose optimization of sirolimus improving drug blood level for seizure control in pediatric patients with TSC.

## Methods

### Patients

The study was approved by the Research Ethics Committee of Children’s Hospital of Fudan University (Ethical code: [2019] 019). In the present study, pediatric patients diagnosed with TSC-related epilepsy from May 2016 to October 2020 at the Children’s Hospital of Fudan University (Shanghai, China) were collected, retrospectively. The criteria for inclusion were as follows: 1) treated by sirolimus, 2) therapeutic drug monitoring for sirolimus. As for the study, it was a retrospective analysis and was approved by the ethics committee of our hospital without the need for written informed consent.

### Therapeutic Drug Monitoring

In the present study, sirolimus was taken once a day and its concentrations were from therapeutic drug monitoring. Blood concentrations were collected prior to the subsequent administration, and the sirolimus concentrations used in the current research were trough concentrations. Sirolimus concentrations were detected using the Emit 2000 Sirolimus Assay (Siemens Healthcare Diagnostics Inc.) with a range of linear response, 3.5–30 ng/ml, whose values of inter-assay variability [coefficient of variation (CV%)] < 4.0%, and values of intra-assay CV (%) < 6.2% (Wang et al., 2020).

### Population Pharmacokinetic Model

The population pharmacokinetic model was established with the nonlinear mixed-effects modeling software, NONMEM (edition 7, ICON Development Solutions, Ellicott City, MD, United States) and a first-order conditional estimation method with interaction (FOCE-I method). A one-compartment model with first-order elimination was used to describe the model because all the concentrations in this research were trough concentrations. The pharmacokinetic parameters, apparent oral clearance (CL/F), volume of distribution (V/F), and absorption rate constant [Ka, fixed at 0.485/h (Wang et al., 2020)], were included.

### Random Effect Model

Inter-individual variabilities were shown in Eq. 1:

$${\text{C}}_{\text{i}}=\text{TV}\left(\text{C}\right)\text{×exp}\left({\text{η}}_{\text{i}}\right)$$ (1)

C_i_, the individual parameter, included CL/F and V/F; TV(C), the typical individual parameter; η_i_, symmetrical distribution, which was a random term with zero mean and variance omega^2 (ω^2^), when available, η_i_ would be added into CL/F and V/F, respectively.

Various random residual variabilities are shown in Eqs. 2–4:

$${\text{O}}_{\text{i}}{\;=\text{ P}}_{\text{i}}{\;+\text{ ε}}_{1}$$ (2)

$${\text{O}}_{\text{i}}{\;=\text{ P}}_{\text{i}}\left({1+\text{ ε}}_{1}\right)$$ (3)

$${\text{O}}_{\text{i}}{\;=\text{ P}}_{\text{i}}\left({1+\text{ ε}}_{1}\right){+\text{ε}}_{2}$$ (4)

${\text{O}}_{\text{i}}$ the observed concentration; ${\text{P}}_{\text{i}}$, the individual predicted concentration; $\varepsilon_{\text{n}}\;$, symmetrical distribution, which was a random term with zero mean and variance sigma^2 (σ^2^), when available, $\varepsilon_{\text{n}}\;$ would be added into ${\text{P}}_{\text{i}}\;$. Eqs. 2–4 are additive error, proportional error, and mixed error, respectively.

### Covariate Model

The relations of pharmacokinetic parameters with weight are shown in Eq. 5:

$${\text{C}}_{\text{i}}{=\text{C}}_{\text{std}}\text{ ×}{\left({\text{W}}_{\text{i}}/{\text{W}}_{\text{std}}\right)}^{\text{power}}$$ (5)

${\text{C}}_{\text{i}}$ the individual parameter; ${\text{W}}_{\text{i}}$ the individual weight; ${\text{W}}_{\text{std}}$ the standard weight of 70 kg; ${\text{C}}_{\text{std}}$, the typical individual parameter, whose weight was W_std_; power, the allometric coefficient: 0.75 for the CL/F and 1 for the V/F (Anderson and Holford, 2008).

Pharmacokinetic parameters and the continuous covariates or categorical covariates are shown in Eqs. 6 Eqs. 7, respectively:

$${\text{C}}_{\text{i}}=\text{TV}\left(\text{C}\right)\text{×}{\left({\text{Cov}}_{\text{i}}/{\text{Cov}}_{\text{median}}\right)}^{\text{θ}}$$ (6)

$${\text{C}}_{\text{i}}=\text{TV}\left(\text{C}\right){\text{×θ}}^{\text{Covi}}$$ (7)

${\text{C}}_{\text{i}}$ the individual parameter; TV(C) the typical individual parameter; θ, the parameter to be estimated; ${\text{Cov}}_{\text{i}}$ the covariate of the i-th individual; ${\text{Cov}}_{\text{median}}$ the population median for the covariate.

The potential covariates included gender, age, weight, sirolimus dosage form, albumin, alanine transaminase, aspartate transaminase, creatinine, urea, total protein, total bile acid, direct bilirubin, total bilibrubin, hematocrit, hemoglobin, mean corpuscular hemoglobin, mean corpuscular hemoglobin concentration, and drug combination. The covariate inclusion criteria were calculated by objective function value (OFV) changes. The decrease of OFV greater than 3.84 (*p* < 0.05) was considered sufficient for inclusion in the base model. The increase of OFV greater than 6.63 (*p* < 0.01) was considered sufficient for significance in the final model.

### Model Evaluation

The goodness-of-fit plots of model (observations *vs.* population predictions, observations *vs.* individual predictions, absolute value of weighted residuals of individual (│iWRES│) *vs.* individual predictions, and weighted residuals *vs.* time), distribution of weighted residuals for model (density *vs.* weighted residuals, and quantilies of weighted residuals *vs.* quantilies of normal), and visual predictive check (VPC) of model were used to estimate the final model. Additionally, the medians and 2.5th–97.5th percentiles of the results from bootstrap (repeated random sampling with replacement from the raw data base with 1,000 repetitions with different random sampling) were used for comparing with final model parameters.

### Simulation

Initial dose optimizations were simulated by the Monte Carlo method, done using the NONMEM software (edition 7, ICON Development Solutions, Ellicott City, MD, United States). According to the report from He *et al*, the target concentration window of sirolimus improving seizure control in pediatric patients with TSC was 5–10 ng/ml (He et al., 2020). In the present study, we found weight, and concomitant medication of oxcarbazepine affected sirolimus clearance. Therefore, on the basis of whether oxcarbazepine was used in combination, and a once-daily sirolimus regimen or a twice-daily sirolimus regimen, we simulated different situations. In each situation, 1,000 virtual pediatric patients were simulated in ten dosages (0.01, 0.02, 0.03, 0.04, 0.05, 0.06, 0.07, 0.08, 0.09, and 0.10 mg/kg/day) for eight weight groups (5, 10, 20, 30, 40, 50, 60, and 70 kg), respectively. The twice-daily sirolimus regimen was split evenly into two dosages a day. The probabilities of achieving the target concentration window were used as the evaluation criteria.

## Results

### Patients

A total of 80 children were included in the present study, 35 boys and 45 girls, aged from 0.61 to 16.61 years. Sirolimus dosage forms included tablet and solution, both dosage forms were used for 41 person-times, respectively (two children used two dosage forms). 188 sirolimus trough concentrations were collected, and the average number of concentrations was 2.35 per patient. The drug combination included carbamazepine, lamotrigine, levetiracetam, oxcarbazepine, topiramate, valproic acid, and vigabatrin. Table 1 shows the demographic data of patients.

**TABLE 1:** Demographic data of patients and the drug combination (n = 80).

Characteristic	Mean ± SD	Median (range)
Gender (boys/girls)	35/45	
Age (years)	6.35 ± 3.83	5.76 (0.61–16.61)
Weight (kg)	23.50 ± 11.71	20.50 (8.00–68.00)
Sirolimus dosage form (tablet/solution)	41/41	two of them used two dosage forms
Albumin (g/L)	43.00 ± 3.41	43.00 (36.00–51.60)
Alanine transaminase (IU/L)	11.67 ± 9.13	10.00 (1.00–50.00)
Aspartate transaminase (IU/L)	28.35 ± 9.23	26.40 (9.00–68.30)
Creatinine (μmol/L)	34.35 ± 10.81	31.55 (20.00–89.00)
Urea (mmol/L)	4.53 ± 1.19	4.50 (2.00–8.30)
Total protein (g/L)	70.71 ± 5.81	70.30 (59.30–80.50)
Total bile acid (μmol/L)	4.03 ± 3.31	3.15 (0.05–14.00)
Direct bilirubin (μmol/L)	2.05 ± 0.78	1.85 (0.70–4.00)
Total bilibrubin (μmol/L)	5.54 ± 2.17	5.10 (2.10–12.60)
Hematocrit (%)	37.66 ± 3.75	37.35 (29.50–49.40)
Hemoglobin (g/L)	125.96 ± 12.51	127.50 (96.00–161.00)
Mean corpuscular hemoglobin (pg)	27.20 ± 1.74	27.20 (23.10–31.00)
Mean corpuscular hemoglobin concentration (g/L)	334.59 ± 11.44	335.00 (305.00–370.00)
Number of co-mediactions		
Carbamazepine	2	
Lamotrigine	4	
Levetiracetam	10	
Oxcarbazepine	23	
Topiramate	8	
Valproic acid	40	
Vigabatrin	12	

### Modeling

In the final model, weight was included as a covariant. In addition, concomitant medication of oxcarbazepine affected sirolimus clearance. Sirolimus dosage forms, or physiological and biochemical indexes were not included in the final model. Hence, the final models were as shown below in Eqs. 8, 9:

$$\text{CL}/\text{F}\;=8\text{.}59\text{×}{\left(\text{weight}/70\right)}^{\text{0}\text{.}75}\text{×}1{\text{.}16}^{\text{OXC}}$$ (8)

V/F=294×(weight/70) (9)

CL/F, apparent oral clearance; V/F, apparent volume of distribution; OXC, oxcarbazepine, when patients received oxcarbazepine, OXC was 1, otherwise OXC was 0.

### Evaluation

**FIGURE 1:**
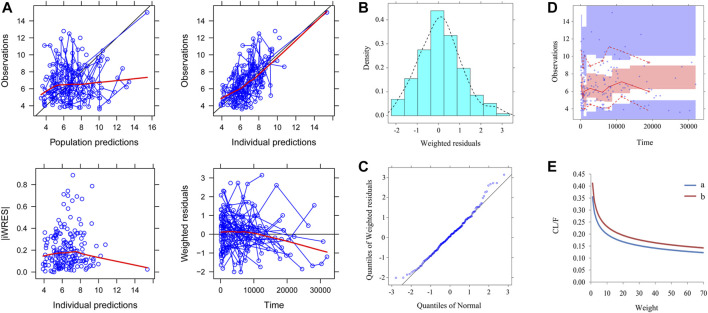
Model evaluation. **(A)** Goodness-of-fit plots of model (observations *vs.* population predictions, observations *vs.* individual predictions, absolute value of weighted residuals of individual (│iWRES│) *vs.* individual predictions, weighted residuals *vs.* time). **(B)** Density *vs.* weighted residuals. **(C)** Quantilies of weighted residuals *vs.* quantilies of normal. **(D)** Visual predictive check (VPC) of model. The middle solid line represents the median of the prediction-corrected concentrations. The lower and upper dashed lines are the 2.5th and 97.5th percentiles of the prediction-corrected concentrations. **(E)** Sirolimus apparent clearance rate (CL/F, L/h/kg) of pediatric patients with different weight, **A**: without oxcarbazepine, **B**: with oxcarbazepine.

Figure 1 shows the final model evaluation. Figure 1A shows goodness-of-fit plots of model. Figures 1B,C show the distribution of weighted residuals. Figure 1D shows the visual predictive check of the model. We found that the distribution of the final model was normal, and most of the observed sirolimus concentrations were within the 95% prediction intervals from the simulation data, showing the prediction-corrected sirolimus concentrations were well predicted by the final model. The parameter estimates of the final model and bootstrap validation are shown in Table 2. The value of inter-individual variability of V/F (ω_V/F_) was very small, close to zero, so we dropped it. In addition, the additive error was selected as the best random residual variability. The median values of 1,000 bootstraps were close to the respective parameter values of the final model, indicating that the model was reliable and accurate.

**TABLE 2:** Parameter estimates of final model and bootstrap validation.

Parameter	Estimate	SE	Bootstrap		Bias (%)
			Median	95% Confidence interval	
CL/F (L/h)	8.59	0.251	8.58	[4.16, 11.30]	−0.12
V/F (L)	294	0.806	292	[58, 973]	−0.85
Ka (h^−1^)	0.485 (fixed)	—	—	—	—
θ_OXC_	1.16	0.062	1.15	[1.03, 1.34]	−0.86
ω_CL/F_	0.175	0.232	0.162	[0.095, 0.246]	−7.43
σ_1_	1.913	0.065	1.895	[1.640, 2.161]	−0.94

95% confidential interval was displayed as the 2.5th, 97.5th percentile of bootstrap estimates. CL/F, apparent oral clearance (L/h); V/F, apparent volume of distribution (L); Ka, absorption rate constant (h^−1^); θ_OXC_ was the coefficient of oxcarbazepine; ω_CL/F_, inter-individual variability of CL/F; σ_1_, residual variability, additive error; bias, prediction error, bias = (median-estimate)/estimate × 100%.

### Simulation

**FIGURE 2:**
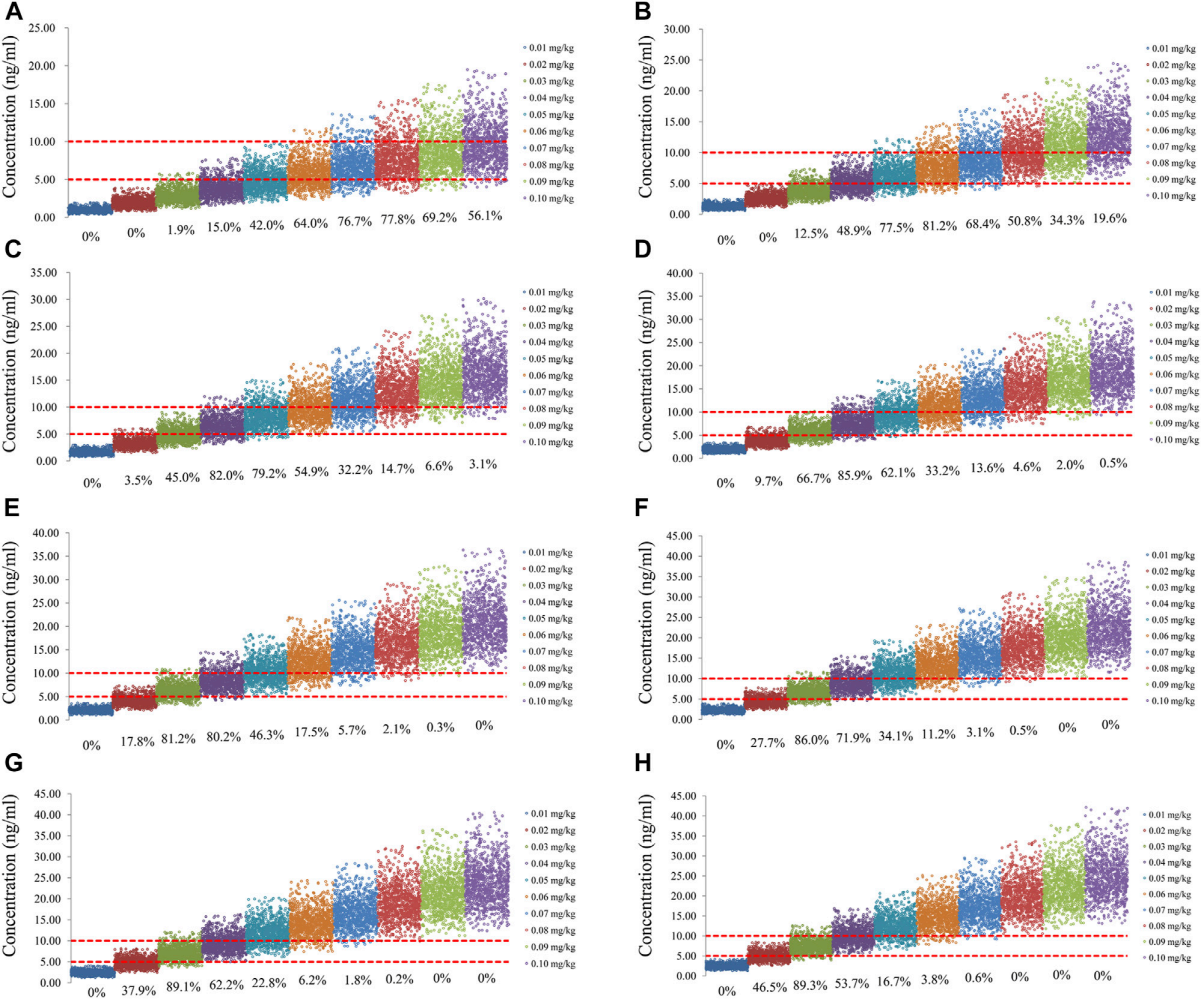
Simulation of sirolimus concentrations of once-daily sirolimus regimen without oxcarbazepine. **(A)** Pediatric patients weighing 5 kg. **(B)** Pediatric patients weighing 10 kg. **(C)** Pediatric patients weighing 20 kg. **(D)** Pediatric patients weighing 30 kg. **(E)** Pediatric patients weighing 40 kg. **(F)** Pediatric patients weighing 50 kg. **(G)** Pediatric patients weighing 60 kg. **(H)** Pediatric patients weighing 70 kg.

**FIGURE 3:**
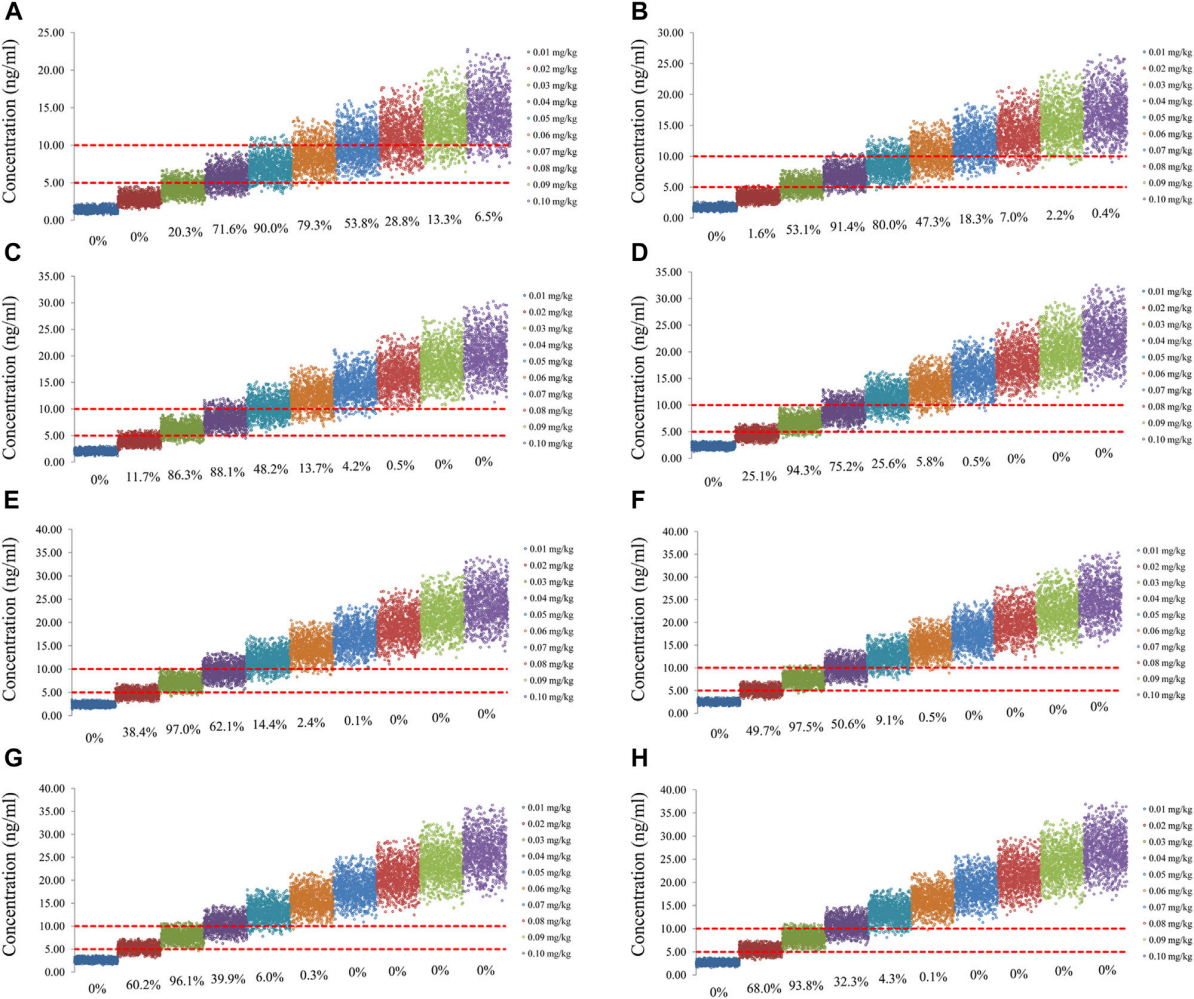
Simulation of sirolimus concentrations of twice-daily sirolimus regimen without oxcarbazepine. **(A)** Pediatric patients weighing 5 kg. **(B)** Pediatric patients weighing 10 kg. **(C)** Pediatric patients weighing 20 kg. **(D)** Pediatric patients weighing 30 kg. **(E)** Pediatric patients weighing 40 kg. **(F)** Pediatric patients weighing 50 kg. **(G)** Pediatric patients weighing 60 kg. **(H)** Pediatric patients weighing 70 kg.

**FIGURE 4:**
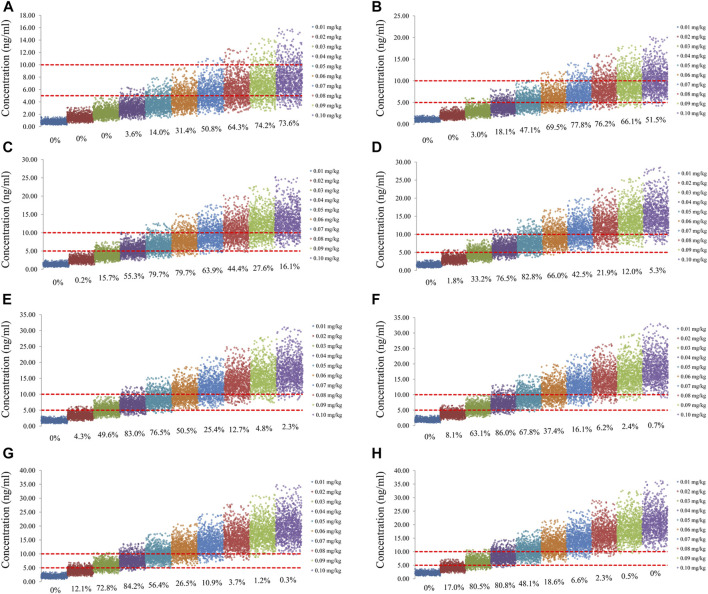
Simulation of sirolimus concentrations of once-daily sirolimus regimen with oxcarbazepine. **(A)** Pediatric patients weighing 5 kg. **(B)** Pediatric patients weighing 10 kg. **(C)** Pediatric patients weighing 20 kg. **(D)** Pediatric patients weighing 30 kg. **(E)** Pediatric patients weighing 40 kg. **(F)** Pediatric patients weighing 50 kg. **(G)** Pediatric patients weighing 60 kg. **(H)** Pediatric patients weighing 70 kg.

**FIGURE 5:**
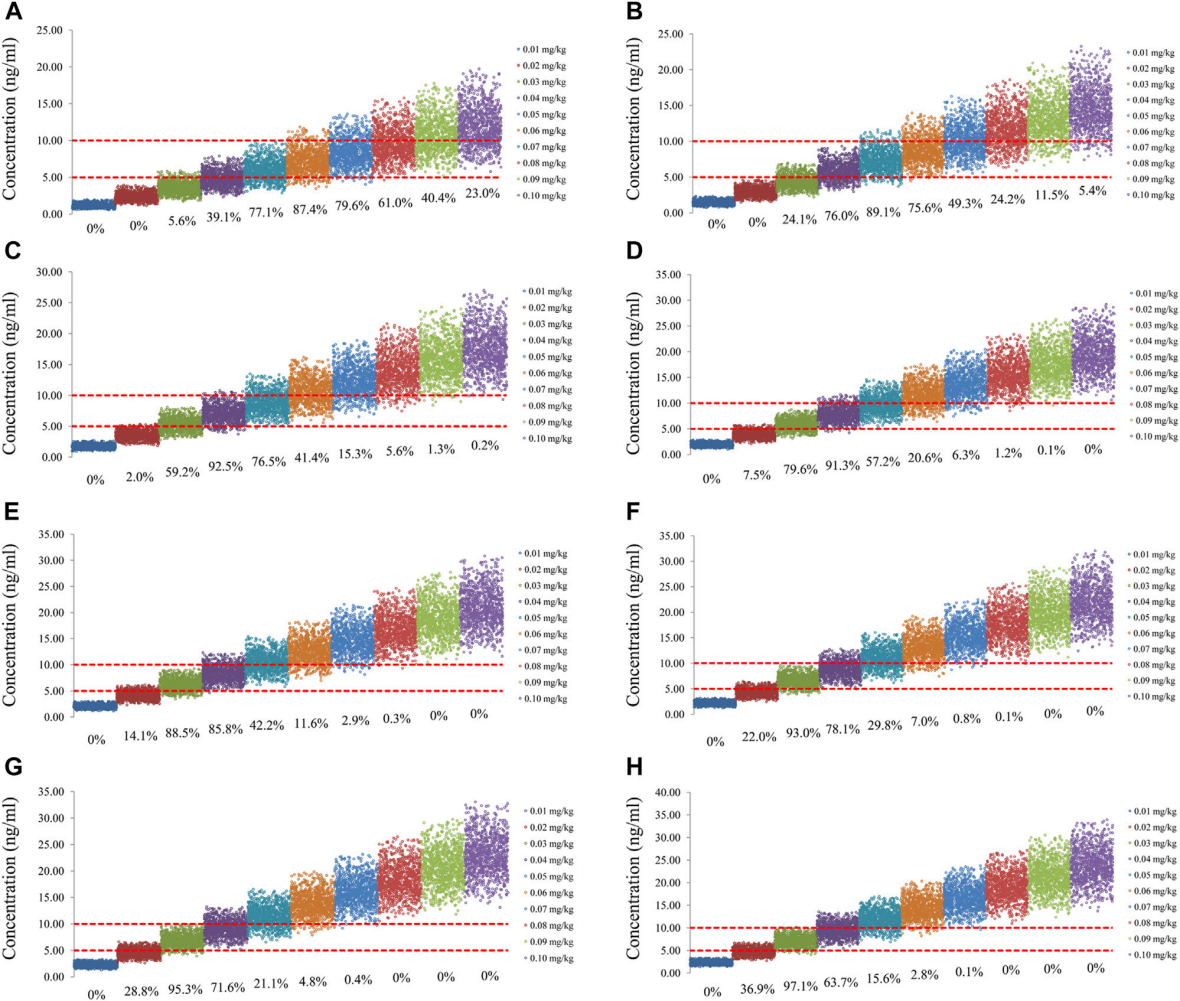
Simulation of sirolimus concentrations of twice-daily sirolimus regimen with oxcarbazepine. **(A)** Pediatric patients weighing 5 kg. **(B)** Pediatric patients weighing 10 kg. **(C)** Pediatric patients weighing 20 kg. **(D)** Pediatric patients weighing 30 kg. **(E)** Pediatric patients weighing 40 kg. **(F)** Pediatric patients weighing 50 kg. **(G)** Pediatric patients weighing 60 kg. **(H)** Pediatric patients weighing 70 kg.

**FIGURE 6:**
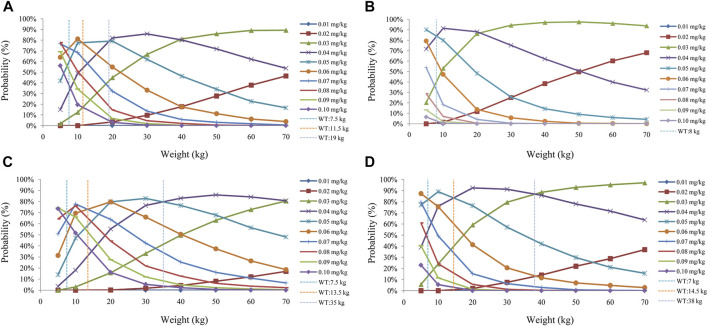
Probability to achieve the target concentrations. **(A)** Once-daily sirolimus regimen without concomitant medication of oxcarbazepine. **(B)** Twice-daily sirolimus regimen without concomitant medication of oxcarbazepine. **(C)** Once-daily sirolimus regimen with concomitant medication of oxcarbazepine. **(D)** Twice-daily sirolimus regimen with concomitant medication of oxcarbazepine.

The sirolimus apparent clearance rate of pediatric patients with different weights are shown in Figure 1E, among which, line a was children without oxcarbazepine and line b was children with oxcarbazepine. With the same weight, the ratios of sirolimus clearance were 1:1.16 in children without oxcarbazepine and children with oxcarbazepine, respectively. Based on the final model, we simulated four different scenarios: 1) without oxcarbazepine, for once-daily sirolimus regimen; 2) without oxcarbazepine, for twice-daily sirolimus regimen; 3) with oxcarbazepine, for once-daily sirolimus regimen; 4) with oxcarbazepine, for twice-daily sirolimus regimen. The simulation of sirolimus concentrations at different initial dosages from the four different scenarios are shown in Figures 2–5, respectively. Figure 6 shows the probabilities of achieving the target concentrations of the four different scenarios; based on this, we recommended the optimal initial administration in each case, which are shown in Table 3. Without oxcarbazepine, for once-daily sirolimus regimen, the doses of 0.07, 0.06, 0.05, 0.04, and 0.03 mg/kg/day were recommended for weights of 5–7.5, 7.5–11.5,11.5–19, 19–40, and 40–70 kg, respectively; for twice-daily sirolimus regimen, the doses of 0.05, 0.04, and 0.03 were recommended for weights of 5–8, 8–20, and 20–70 kg, respectively. With oxcarbazepine, for once-daily sirolimus regimen, the doses of 0.09, 0.08, 0.07, 0.06, 0.05, and 0.04 mg/kg/day were recommended for weights of 5–7.5, 7.5–10, 10–13.5, 13.5–20, 20–35, and 35–70 kg, respectively; for twice-daily sirolimus regimen, the doses of 0.06, 0.05, 0.04, and 0.03 were recommended for weights of 5–7, 7–14.5, 14.5–38, and 38–70 kg, respectively.

**TABLE 3:** Initial dosage recommendation of sirolimus.

Without oxcarbazepine				With oxcarbazepine			
Once a day		Split evenly into two doses a day		Once a day		Split evenly into two doses a day	
Body weight (kg)	Dose (mg/kg/day)	Body weight (kg)	Dose (mg/kg/day)	Body weight (kg)	Dose (mg/kg/day)	Body weight (kg)	Dose (mg/kg/day)
5–7.5	0.07	5–8	0.05	5–7.5	0.09	5–7	0.06
7.5–11.5	0.06	8–20	0.04	7.5–10	0.08	7–14.5	0.05
11.5–19	0.05	20–70	0.03	10–13.5	0.07	14.5–38	0.04
19–40	0.04			13.5–20	0.06	38–70	0.03
40–70	0.03			20–35	0.05		
				35–70	0.04		

## Discussion

It is verified that mTOR inhibitors have good effects on treating TSC (Palavra et al., 2017; Franz and Krueger, 2018; Li et al., 2019). Krueger *et al* reported short-term safety of mTOR inhibitors in infants and very young children with TSC (Krueger et al., 2018). Saffari *et al* reported the safety and efficacy of mTOR inhibitor treatment in patients with TSC under 2 years of age (Saffari et al., 2019). Among them, everolimus is a registered mTOR inhibitor for epilepsy in TSC. French *et al* reported adjunctive everolimus treatment significantly reduced seizure frequency with a tolerable safety profile compared with placebo in patients with TSC and treatment-resistant seizures (French et al., 2016). In addition, He *et al* reported that sirolimus has a significant effect on seizures associated with TSC, and it could be used as the first-line medication for pediatric patients with TSC-associated epilepsy and the target concentration window of sirolimus was 5–10 ng/ml (He et al., 2020).

In the traditional clinical diagnosis and treatment process, sirolimus concentrations were monitored using therapeutic drug monitoring, subsequently adjusting the next sirolimus dosage based on concentrations. However, the initial dosage could not be recommended by the method. Luckily, the combination of population pharmacokinetics and Monte Carlo simulation could be used to recommend the optimum initial regimen, which has been successfully used in many drugs. For example, Zhao *et al* reported developmental population pharmacokinetics and dosing optimization of cefepime in neonates and young infants (Zhao et al., 2020). Therefore, the present study aimed to recommend the initial dosage regimen for sirolimus improving drug blood level for seizure control in pediatric patients with TSC based on the population pharmacokinetics and Monte Carlo simulation.

In our study, weight and concomitant medication of oxcarbazepine affected sirolimus clearance. It has been reported that there was a nonlinear relationship between drug clearance and weight in pediatric patients, which may be well described by allometric scaling with the coefficients of 0.75 for clearance and 1 for volume (Anderson and Holford, 2008; Hao et al., 2018). In addition, oxcarbazepine is a cytochrome P450 (CYP) 3A4 inducer (McGrane et al., 2018) and sirolimus is primarily metabolized by the cytochrome CYP3A4 and CYP3A5 (Zhang et al., 2017). Oxcarbazepine may accelerate sirolimus metabolism by inducing 3A4 activity. And at the same weight, the ratios of sirolimus clearance were 1:1.16 from children without oxcarbazepine and children with oxcarbazepine, respectively. These results suggested that the dose of sirolimus should be increased when oxcarbazepine was included in the epilepsy regimen. In addition, carbamazepine is also an inducer of CYP3A4. Theoretically, it may have potential interaction with sirolimus. However, carbamazepine could not be included as a statistically significant covariable in the present study. What is interesting about this is that it is not isolated, *Chen et al* reported evaluation of CYP3A4-based interactions of levomilnacipran with carbamazepine in healthy subjects, also no dose adjustment for levomilnacipran is suggested when levomilnacipran is co-administered with carbamazepine (Chen et al., 2015). We also found that sirolimus dosage forms, tablet or solution, had no significant effect on sirolimus clearance rate, suggesting that dosage forms suitable for specific age-groups of children can be selected according to the actual clinical needs.

Ultimately, based on the final model, we recommended the optimal initial administration in each case. Without oxcarbazepine, for once-daily sirolimus regimen, the doses of 0.07, 0.06, 0.05, 0.04, and 0.03 mg/kg/day were recommended for weights of 5–7.5, 7.5–11.5,11.5–19, 19–40, and 40–70 kg, respectively; for twice-daily sirolimus regimen, the doses of 0.05, 0.04, and 0.03 were recommended for weights of 5–8, 8–20, and 20–70 kg, respectively. With oxcarbazepine, for once-daily sirolimus regimen, the doses of 0.09, 0.08, 0.07, 0.06, 0.05, and 0.04 mg/kg/day were recommended for weights of 5–7.5, 7.5–10, 10–13.5, 13.5–20, 20–35, and 35–70 kg, respectively; for twice-daily sirolimus regimen, the doses of 0.06, 0.05, 0.04, and 0.03 were recommended for weights of 5–7, 7–14.5, 14.5–38, and 38–70 kg, respectively. In addition, we found that in the same situation, the twice-daily sirolimus regimen had higher probabilities of achieving the target concentrations, meanwhile reduced total use of sirolimus, which could reduce the medical cost to some extent. But, the downside was that it may increase the frequency of use and may decrease medication compliance in patients. Hence, the clinician or pharmacist should choose the specific selection of once-daily or twice-daily sirolimus regimen based on the actual clinical situation.

Of course, to some extent, *CYP3A4* and *CYP3A5* may influence sirolimus metabolism in adults (Tamashiro et al., 2017; Zhang et al., 2017). However, it was interesting in our previous study that the initial dosage recommendation for sirolimus in children with TSC, *CYP3A4*, and *CYP3A5* polymorphism did not significantly affect sirolimus clearance rates (Wang et al., 2020). The main reason was that included children had a wide age span (Wang et al., 2020). And it was reported that gene expression in different age development processes had development-dependent characteristics; meanwhile, CYP3A expression isoforms levels were highly variable after birth (Lacroix et al., 1997). That is to say, the activity of drug-metabolizing enzymes of the same CYP3A genotype may be different in different age-groups and genotypes may not accurately explain the differences from sirolimus concentrations. Other studies had also confirmed that CYP3A genotypes were not included in population pharmacokinetics of sirolimus for children (Mizuno et al., 2017a; Mizuno et al., 2017b). Most important of all, the sirolimus pharmacogenomics was not tested routinely in current sirolimus therapeutics, and the weight-based dosage recommendations had more convenient and better clinical application advantages (Wang et al., 2020). Based on the above, the current study did not further examine the influence of *CYP3A4* and *CYP3A5* genotypes. However, further studies will be conducted on the newly identified polymorphism that may significantly affect sirolimus clearance in pediatric patients. In addition, it was an objective existence that sparse clinical sampling mostly contained insufficient information, which was also a limitation of our study. We will further verify the conclusion of our study through prospective intensive sampling in the future.

## Conclusion

The present study was the first to establish a population pharmacokinetic model of sirolimus improving drug blood level for seizure control in pediatric patients with TSC and recommend the initial dosage regimen.

## Data Availability

The original contributions presented in the study are included in the article/Supplementary Material, further inquiries can be directed to the corresponding authors.
